# Emerging information technologies across the autism service and prevention continuum: a narrative review of screening, diagnosis, intervention, and health equity

**DOI:** 10.3389/fpubh.2026.1900591

**Published:** 2026-09-28

**Authors:** Xugao Han, Zhen Liu, Houxi Xu, Lingyan Weng

**Affiliations:** 1 School of Medical Humanities, Nanjing University of Chinese Medicine, Nanjing, China; 2 Key Laboratory of Acupuncture and Medicine Research of Ministry of Education, Nanjing University of Chinese Medicine, Nanjing, China; 3 School of Tourism Management, Nanjing Institute of Tourism and Hospitality, Nanjing, China

**Keywords:** artificial intelligence, autism spectrum disorder, digital health, early screening, health equity, public health, telehealth, virtual reality

## Abstract

Autism spectrum disorder (ASD) is a major global public-health concern, yet timely identification and support remain difficult to access for many families, especially in rural, low-resource, and otherwise underserved settings. Emerging information technologies have been proposed as tools for narrowing this service gap. These include artificial intelligence (AI), wearable sensors, and the Internet of Things, mobile health applications and digital therapeutics, telehealth, immersive virtual and augmented reality, social robotics, and large language models. This narrative review organizes the evidence across an autism service and prevention continuum: upstream risk awareness and health education, early screening and diagnosis, and intervention, management, and long-term support. We use this continuum strictly as a public-health service-organization framework, not to imply preventing the birth or existence of autistic individuals, consistent with a neurodiversity-affirming perspective. Across each stage, we apply a health-equity lens centered on reach, affordability, scalability, health-system integration, and equity of performance. We summarize current applications, appraise recurring limitations, and synthesize cross-cutting issues of equity, data governance, ethics, neurodiversity, and implementation. Recurring limitations include small and heterogeneous studies, dataset bias, weak external validation, short follow-up, and uneven regulatory maturity. Emerging information technologies show genuine promise as complements to conventional autism services, particularly where specialist resources are scarce. Realizing population-level benefit will require equity-centered design, validation in diverse and low-resource settings, larger and longer trials, interoperable standards, and meaningful involvement of autistic people and their families.

## 1 Introduction

Autism spectrum disorder (ASD) is a lifelong neurodevelopmental condition characterized by differences in social communication and interaction, together with restricted, repetitive patterns of behavior and interests. Once considered rare, ASD is now understood to be common and to constitute a substantial and growing public-health burden worldwide. Estimates from the Global Burden of Disease (GBD) Study 2021 indicate that approximately 61.8 million people, about one in every 127 individuals, were on the autism spectrum globally in 2021, and that ASD accounted for roughly 11.5 million disability-adjusted life-years, ranking within the top ten causes of non-fatal health burden among people younger than 20 years (1). Complementary GBD analyses place neurodevelopmental and other disorders affecting the nervous system among the leading contributors to global disease burden, with ASD specifically identified among the ten conditions with the highest age-standardized burden (2), and document the persistent and rising contribution of mental and neurodevelopmental disorders to population health loss over recent decades (3, 4). These figures establish ASD not merely as a clinical entity managed at the level of the individual, but as a population-level concern that demands public-health strategies for prevention, early identification, and support.

A defining feature of the autism field is the gap between what is known to improve outcomes and what most affected children and families can actually access. There is broad consensus that early identification and intervention, delivered when neural plasticity is greatest, are associated with better developmental trajectories (5, 6). Yet the conventional diagnostic pathway depends on lengthy, specialist-administered behavioral assessments that are time-consuming, partly subjective, and concentrated in urban centers and high-income settings (5, 7). The consequences are long waiting lists, late diagnosis, and pronounced disparities: children in rural areas, in low- and middle-income countries (LMICs), and from marginalized or socioeconomically disadvantaged communities are systematically underdiagnosed and underserved (8), while families in better-resourced settings increasingly access early screening and intervention (9). The result is a global “autism service gap”: measured prevalence and recorded burden are strongly shaped by diagnostic capacity, while under-ascertainment, under-diagnosis, and limited access to trained professionals and validated tools remain especially pronounced in LMICs and other under-resourced settings (1, 9).

Against this backdrop, emerging information technologies have attracted intense interest as potential tools for narrowing the autism service gap. Advances in artificial intelligence (AI) and machine learning, the proliferation of low-cost sensors and wearable devices, the maturation of telehealth, the spread of smartphones even in resource-limited settings, and the emergence of immersive media and conversational AI have created new opportunities to extend screening, diagnosis, intervention, and long-term support beyond conventional service models (10, 11). However, these technologies differ substantially in their scalability and resource requirements. Community-oriented approaches, such as telehealth and app- or video-based screening, may reduce dependence on geographically concentrated specialist services and expand access in underserved settings, whereas neurophysiological, biomarker-based, and other resource-intensive approaches may require specialized equipment, technical expertise, and infrastructure. Across this heterogeneous technological ecosystem, potential benefits must therefore be weighed against risks of bias, widening rather than narrowing inequities, premature clinical adoption ahead of robust validation, and insufficient attention to the priorities of autistic people themselves.

Against this background, the present narrative review brings together evidence on emerging information technologies across the autism service and prevention continuum from a public-health and health-equity perspective. Specifically, this review has four main objectives: (1) to map the current applications of emerging information technologies across upstream public-health action, early screening and diagnosis, and intervention, management, and long-term support; (2) to critically appraise the strength, consistency, and major limitations of the evidence supporting these technologies; (3) to evaluate their public-health relevance using five equity-oriented criteria—reach, affordability, scalability, health-system integration, and equity of performance; and (4) to identify the ethical, regulatory, data-governance, neurodiversity, and implementation issues that may determine whether these technologies reduce or widen existing disparities in autism-related services.

To address these objectives, the review was guided by three research questions. First, how are emerging information technologies currently applied across upstream public-health action, early screening and diagnosis, and intervention, management, and long-term support for autistic individuals? Second, what are the principal strengths, limitations, and levels of evidence supporting these technologies across the different stages of the autism service and prevention continuum? Third, under what conditions are these technologies likely to reduce, reproduce, or widen inequities in access, affordability, scalability, health-system integration, and performance across different populations and settings?

The distinctive contribution of this review is not simply to describe individual technologies, many of which have already been examined in technology- or stage-specific reviews (12–18). Rather, it integrates a fragmented evidence base within a single public-health service continuum and evaluates these technologies using a consistent health-equity framework. This approach shifts the central question from technical performance alone to whether a technology expands access, for whom it works, in which settings it can be implemented, and at what cost. The prevention continuum is used solely as a framework for organizing evidence on developmental surveillance, early identification, intervention, and support. It is not intended to imply that autism or autistic individuals should be prevented, but rather to examine how timely, accessible, and equitable support can be strengthened across different stages of service delivery.

## 2 Conceptual framework

### 2.1 Defining “emerging information technologies”

For this review, “emerging information technologies” refers to digital and computational tools whose application to autism care has expanded rapidly over the past decade. These include AI and machine learning, wearable sensors and the Internet of Things (IoT), mobile health (mHealth) applications and digital therapeutics, telehealth and telemedicine, virtual and augmented reality (VR/AR), social and assistive robotics, and large language models (LLMs) or other forms of generative AI. These categories frequently overlap: wearables generate data analyzed by machine learning, telehealth platforms can host digital therapeutics, and robots may integrate computer vision or speech analysis. In practice, the most promising systems are increasingly multimodal (10, 19).

### 2.2 The autism service and prevention continuum

We adopt the public-health framework of primary, secondary, and tertiary prevention as the organizing spine of this review, while explicitly adapting it to the autism context. Autism is not a disease to be eradicated, and the language of “prevention and control,” when applied uncritically, risks stigmatization and conflicts with a neurodiversity-affirming perspective that regards autism as a form of human variation. We therefore use the prevention continuum descriptively and solely as a service-organization framework. In particular, “primary prevention” in this review does not refer to preventing the birth or existence of autistic individuals. Rather, it refers to upstream public-health activities such as attention to modifiable risk factors for adverse outcomes, population-level awareness, developmental health literacy, and risk-informed surveillance that can expand timely access to support.

Within this adapted framework, primary prevention denotes upstream risk awareness, health education, and population-level developmental surveillance; secondary prevention denotes early screening and timely, accurate diagnosis so that support can begin during periods of peak developmental plasticity (5, 6); and tertiary prevention denotes intervention, management, and long-term support intended to optimize functioning, participation, and quality of life rather than to “cure.” Throughout, we treat early identification and support as goods because they expand options and access to services, while remaining attentive to the risks of labeling, over-diagnosis, and loss of autonomy that the literature also documents (5, 7). Where risk-oriented technologies are discussed in Section 4.1, they are framed as tools for risk-informed surveillance and education, explicitly not as instruments of selection.

### 2.3 Public-health and health-equity lens

To evaluate technologies as public-health tools rather than as clinical novelties, we apply five equity-oriented criteria consistently across the continuum: reach (can the tool identify or serve populations currently missed by conventional services?), affordability (is the cost compatible with deployment at scale, including in resource-limited settings?), scalability (can it function without proportional increases in scarce specialist labor?), health-system integration (can it be embedded into existing health systems, primary care, and community services?), and equity of performance (does it work as well across sexes, ethnicities, languages, and geographies, or does it encode and amplify existing disparities?). These criteria draw on widely used frameworks for healthcare access, digital health equity, and the fair deployment of AI in medicine (9, 20, 21), and they structure the critical appraisal in each section that follows. Figure 1 summarizes the conceptual framework used in this review, positioning emerging information technologies across the autism service and prevention continuum and showing the health-equity lens applied throughout.

**Figure 1:**
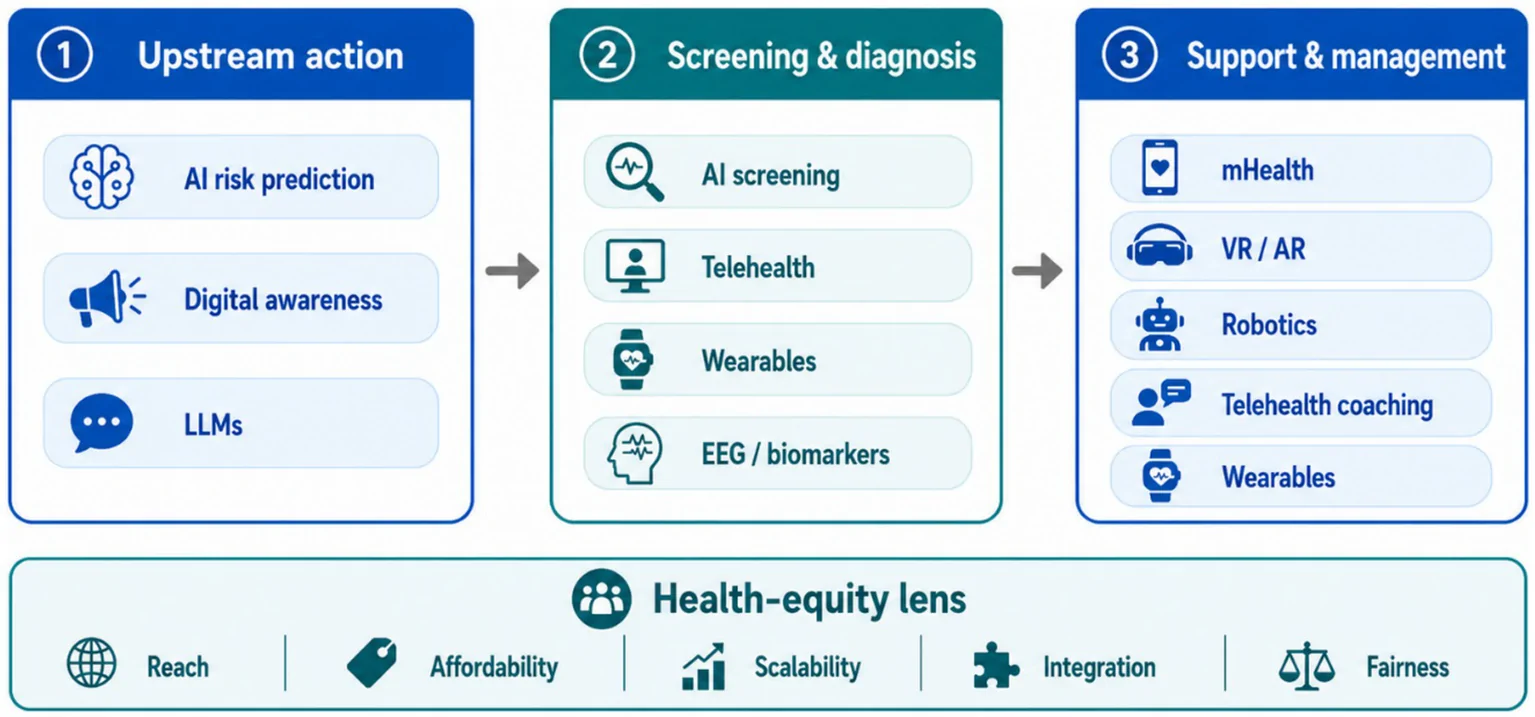
Conceptual framework of the autism service and prevention continuum. The figure positions emerging information technologies across three stages of the adapted public-health continuum: upstream action, screening and diagnosis, and support and management. The continuum is used as a service-organization framework rather than to imply that autism or autistic people should be prevented. The health-equity lens consists of five criteria: reach, affordability, scalability, health-system integration, and equity of performance. These criteria apply across all stages.

## 3 Review methodology

### 3.1 Review design

This review was conducted as a narrative review rather than a systematic review. Its purpose was to provide a structured and critical synthesis of clinically and publicly relevant evidence on emerging information technologies across the autism service and prevention continuum. The review did not seek exhaustive, protocol-driven coverage of all technical studies or undertake a formal certainty-of-evidence assessment. Nevertheless, we specified the information source, search strategy, eligibility criteria, and analytical approach to improve transparency and reduce the risk of selective citation.

### 3.2 Information source and search strategy

PubMed was used as the sole searchable bibliographic database. It was selected because the central focus of the review was the clinical and public-health application of emerging information technologies in autism-related developmental surveillance, screening, diagnosis, intervention, long-term support, health equity, ethics, and implementation. PubMed provides broad coverage of peer-reviewed research in medicine, public health, psychiatry, pediatrics, neuroscience, rehabilitation, digital health, and health-services research and was therefore considered appropriate for the clinical and population-health orientation of this review.

The review was not intended to provide an exhaustive account of all technical developments in engineering, computer science, or human–computer interaction. Rather, it focused on technologies with a clear connection to autism-related healthcare, developmental or functional outcomes, service delivery, accessibility, or public-health implementation. Purely technical studies addressing only algorithm development, software architecture, device engineering, or computational performance, without a clearly described clinical, behavioral, service-related, or public-health application, were therefore outside the principal scope of the review.

The principal search period covered publications from January 2022 through December 2025, and the search was last updated in December 2025. This period was selected because of the rapid development of artificial intelligence, digital health, wearable technologies, immersive technologies, and generative AI. Earlier publications were retained selectively when they were necessary to support established concepts, including the global burden of autism, developmental plasticity, neurodiversity, health-equity frameworks, ethical principles, and health-technology implementation models.

Searches combined autism-related terms (“autism,” “autism spectrum disorder,” and “ASD”) with technology-related terms, including “artificial intelligence,” “machine learning,” “computer vision,” “natural language processing,” “wearable,” “sensor,” “Internet of Things,” “mobile health,” “digital intervention,” “digital therapeutic,” “telehealth,” “telemedicine,” “virtual reality,” “augmented reality,” “robot,” “socially assistive robot,” “large language model,” and “generative AI.” These terms were combined, where appropriate, with service-stage and outcome terms such as “risk,” “developmental surveillance,” “screening,” “diagnosis,” “intervention,” “management,” “long-term support,” “access,” “equity,” “disparity,” “bias,” “ethics,” “privacy,” and “implementation.”

### 3.3 Eligibility criteria

Peer-reviewed, full-text, English-language publications were considered eligible when they: (1) focused on autistic individuals, individuals undergoing autism-related screening or assessment, caregivers, healthcare professionals, or autism-related service systems; (2) examined an emerging information technology relevant to risk awareness, developmental surveillance, screening, diagnosis, intervention, management, service delivery, or long-term support; and (3) reported findings relevant to clinical practice, behavioral or functional outcomes, accessibility, health equity, ethics, data governance, regulation, usability, or implementation.

Eligible publication types included quantitative, qualitative, and mixed-methods primary studies; diagnostic-accuracy studies; randomized and nonrandomized intervention studies; observational studies; systematic reviews; scoping reviews; and meta-analyses. Directly relevant methodological, conceptual, and implementation publications were also retained when they informed the review’s public-health, ethical, equity, or health-system analysis. Older foundational publications were included when required to support stable concepts rather than current claims concerning the performance of specific technologies.

Publications were excluded when they: (1) did not focus on autism or did not report autism-specific findings separately; (2) described a technology without a clear clinical, behavioral, service-delivery, accessibility, or public-health connection; (3) were conference abstracts without a full peer-reviewed publication; (4) were editorials, letters, or opinion articles without substantive empirical evidence or structured conceptual analysis; (5) were duplicate publications; (6) were not published in English; or (7) presented unpublished or non-peer-reviewed material.

### 3.4 Evidence organization and analytical approach

The literature was organized and synthesized in accordance with the objectives and research questions stated in the Introduction. The analytical process consisted of three related stages. First, to map the applications of emerging information technologies across the autism service continuum, eligible evidence was grouped according to three stages of the adapted framework: upstream public-health action, early screening and diagnosis, and intervention, management, and long-term support. Within each stage, publications were further organized by technology category, including artificial intelligence and machine learning, mobile health and digital therapeutics, wearable and sensor technologies, telehealth, immersive technologies, social and assistive robotics, and generative AI. Second, to qualitatively evaluate the maturity, strengths, and limitations of the available evidence, we considered, where applicable and as reported in the included literature, study design, sample size, comparator selection, reported effectiveness or diagnostic performance, external validation, follow-up duration, population representativeness, and real-world applicability. Particular attention was given to differences between proof-of-concept findings and evidence sufficient to support routine clinical or public-health implementation. Recurring limitations, including small and heterogeneous samples, curated datasets, limited external validation, short follow-up, and weak comparative-effectiveness evidence, were identified across technology categories. Third, to examine the public-health and health-equity implications of these technologies, the evidence was appraised using the five criteria defined in Section 2.3: reach, affordability, scalability, health-system integration, and equity of performance. Cross-cutting issues related to digital access, usability, ethics, neurodiversity, data governance, privacy, regulation, financing, and implementation were also examined.

No new pooled effect estimates were calculated. Where quantitative summary estimates are presented, they were derived from previously published systematic reviews or meta-analyses. The resulting synthesis should therefore be interpreted as a structured and critical narrative map of the field rather than as a formal systematic review, risk-of-bias assessment, or certainty-of-evidence grading.

## 4 Evidence synthesis across the autism service and prevention continuum

In accordance with the analytical approach described in Section 3.4, the evidence is presented across three stages of the adapted autism service and prevention continuum. These stages are used to organize the findings according to the principal public-health and service function of each technology rather than to represent separate methodological approaches. Some technologies operate across more than one stage; in such cases, they are discussed in the section corresponding to their predominant application, with cross-stage implications considered in Section 5.

### 4.1 Upstream public-health action: risk awareness and developmental surveillance

This section corresponds to the primary-prevention component of the adapted framework described above, but frames it as upstream public-health action to avoid implying that autism itself should be prevented. In the autism context, primary prevention is therefore used in an upstream public-health sense rather than in a deterministic or etiological sense. It encompasses attention to modifiable risk factors for adverse outcomes, early flagging of elevated likelihood before symptoms prompt clinical referral, and digital channels for population awareness and health literacy. Some risk-stratification tools discussed below sit at the boundary between population awareness, developmental surveillance, and early identification; they are therefore interpreted as mechanisms for expanding timely access to support, not as instruments for preventing autism itself.

#### 4.1.1 Data-driven risk identification

The increasing availability of large clinical and population datasets has enabled machine-learning approaches to risk stratification. A notable example is the use of machine-learning models trained on a minimal set of routinely collected background and early medical-history items to predict the likelihood of an eventual ASD diagnosis. In one large study drawing on a research cohort of tens of thousands of participants, gradient-boosting models using a small number of early-life items, including developmental milestones and early eating behavior, achieved good discrimination for identifying autistic individuals and retained reasonable performance on independent validation cohorts (19). From a public-health standpoint, the appeal of such approaches is that they rely on information that can be captured cheaply and early, potentially during routine well-child contacts, and that explainable-AI methods can surface which factors drive predictions, supporting clinician interpretability (19, 22). Nevertheless, this line of work illustrates a broader public-health tension: even models that perform well in research cohorts require prospective testing in routine, demographically diverse populations before being used for population-level risk stratification. Predictive screening also carries the risk of false positives and consequent family anxiety.

#### 4.1.2 Biological risk markers and the limits of “prevention”

A parallel line of work examines biological markers, including genetic, metabolic, trace-element, and gut-microbiome differences, as potential indicators of ASD risk. A meta-analysis comparing autistic children with controls reported group-level differences in several biomedical markers, trace elements, and microbiota measures, suggesting candidate biological signatures that might 1 day contribute to earlier, biologically grounded risk identification (23). Yet these are group-level associations with substantial heterogeneity; none is presently a validated, deployable population screening tool, and the framing of biological “risk” for a neurodevelopmental difference raises ethical questions. Surveys of parental perspectives on early-life and genetic screening show that many caregivers favor earlier identification and would value earlier access to support, while also needing clearer information and counseling, underscoring that risk-oriented technologies must be embedded in compassionate, well-informed communication rather than deployed as standalone tests (7).

#### 4.1.3 Digital awareness, education, and health literacy

Beyond formal risk modeling, emerging information technologies shape primary prevention through population awareness. Smartphones, social media, web platforms, and increasingly conversational AI are now major sources of health information for parents, and improved awareness of early developmental “red flags” is itself a driver of timely help-seeking (5). Generative AI tools such as large language models are being explored as accessible, low-cost sources of health information and triage support. Their promise is scalability; their risks lie in inaccuracy and inequity. A revealing study of an LLM’s responses to common patient questions found that answers generated in a non-English language scored significantly lower for accuracy than English answers, illustrating how generative AI can inadvertently extend existing health inequities and propagate misinformation among linguistic-minority populations (24). For autism specifically, where caregiver knowledge and stigma strongly shape help-seeking, digital health-education tools could expand reach, but only if their accuracy is validated across the languages and cultures of the populations they are meant to serve.

#### 4.1.4 Public-health appraisal

Judged against our equity criteria, technology-enabled primary prevention is the least mature stage of the continuum. Data-driven risk models score well on potential reach and affordability but remain unproven at the population scale and vulnerable to bias; biological markers remain pre-translational; and digital awareness tools are highly scalable but carry acute accuracy and equity risks. Importantly, primary-prevention applications should be framed as risk-informed surveillance and education that expand access to support, explicitly avoiding any deterministic or eugenic framing, a boundary that is both an ethical imperative and a precondition for community trust.

### 4.2 Early screening and diagnosis

Secondary prevention refers here to early, accurate screening and diagnosis. This is the stage at which emerging information technologies have been studied most intensively, reflecting both the clarity of the problem (long waits, scarce specialists, subjective assessment) and the suitability of autism’s observable behavioral and physiological features for automated analysis.

#### 4.2.1 AI-based behavioral and developmental screening

A large body of work applies AI and machine learning to behavioral signals that distinguish autistic from non-autistic children. A systematic review of AI-based screening organized the relevant markers into five categories: gaze behavior, facial expressions, motor movements, voice features, and task performance. Across studies, AI screening achieved accuracies ranging from roughly 62 to 100%, with correspondingly wide ranges in sensitivity and specificity (12). This range is itself the key message: under favorable, often constrained conditions, automated systems can match or exceed conventional screening, but performance is highly variable and dependent on dataset and setting. Computer-vision analysis of children’s gaze and facial expression, automated coding of motor and play behavior, and speech/natural-language analysis of vocalizations are all active approaches, and the same review concludes that multimodal screening, combining several marker types, is the most promising route to robust, generalizable accuracy (12). Methodological studies continue to refine these systems; for example, work combining classical classifiers with explainable-AI feature selection reported high classification accuracy on toddler screening datasets while also attempting to tailor downstream educational strategies to the individual child (22). The public-health attraction is clear: such tools could enable low-cost, large-scale, app-delivered first-stage screening administered by non-specialists or caregivers. A further public-health caveat is that high accuracy in enriched or case–control datasets may not translate directly into population screening. In low-prevalence settings, even models with apparently strong sensitivity and specificity may generate substantial false-positive referrals, making positive predictive value, referral burden, and downstream service capacity as important as headline accuracy.

#### 4.2.2 Neurophysiological and biomarker-based approaches

Complementary approaches seek more biologically grounded signals. Electroencephalography (EEG) combined with machine learning has been reviewed as a route to objective ASD identification, with studies reporting that interpretable neural features can be extracted and classified, although the field is constrained by small, heterogeneous datasets and limited standardization of acquisition and preprocessing (13). As noted above, biomarker panels spanning metabolic, immune, trace-element, and microbiome measures show group-level differences between autistic and non-autistic children and have been proposed as candidate diagnostic contributors (23). The integration of AI with such biomarkers, “hybrid” models balancing accessibility and biological specificity, is frequently advanced as a future direction, but at present, these methods remain predominantly research tools rather than validated, scalable screening instruments. From an equity perspective, these biologically oriented approaches also risk privileging settings with specialized equipment, laboratory capacity, and technical expertise. Unless acquisition protocols, analytic pipelines, and normative datasets are standardized across diverse populations, they may add objectivity in research settings without improving population-level access (13, 23).

#### 4.2.3 Wearables, sensors, and the internet of things

Wearable and sensor technologies extend screening and characterization beyond the clinic into everyday environments. Reviews of wearable devices in ASD describe their use to capture physiological signals such as heart rate, heart-rate variability, electrodermal activity, and movement. Common sensors include electrocardiography (ECG), electroencephalography (EEG), photoplethysmography (PPG), and electromyography (EMG), with applications spanning stress and arousal monitoring, behavioral characterization, and safety (14, 25, 26). A systematic review of wearable solutions for stress monitoring in autistic individuals found that most studies relied on cardiac signals captured by photoplethysmography and electrocardiography, but highlighted small sample sizes, variability across study conditions, and the need to individualize stress-detection thresholds because of high inter-person variability (14). A broad review of commercial wearables for autism management cataloged 110 devices and reported that a majority had undefined US Food and Drug Administration (FDA) regulatory status, with only a minority having received clearance. This is an important caution for public-health deployment, particularly because FDA status reflects one national regulatory context rather than a global standard (25). Reviews of wearable sensors for safety during physical activity similarly emphasize promise alongside unresolved issues of device usability, data privacy, and real-world validation (26). Sensor accuracy is itself non-trivial: head-to-head evaluation of a consumer wearable against polysomnography for sleep classification, relevant given the high prevalence of sleep difficulties in autism, showed reasonable performance but also the limits of consumer devices for clinical-grade measurement (27). A recurrent and autism-specific design constraint is sensory sensitivity: devices that are uncomfortable or intolerable will not be worn, so user-centered, unobtrusive design is not a luxury but a determinant of validity (14, 25).

#### 4.2.4 Telehealth-enabled remote screening and diagnosis

Perhaps the most directly equity-relevant secondary-prevention technology is telehealth, which decouples assessment from the physical location of scarce specialists. A literature review of the equivalence between telemedicine and face-to-face diagnosis of ASD in children concluded that validated remote tools for first-level screening, structured assessment scales, and diagnostic procedures can achieve high specificity and sensitivity with good family satisfaction, supporting timelier detection (15). A 2025 systematic review and meta-analysis of video-assisted telehealth for autism screening and diagnosis pooled 41 studies and reported good sensitivity (around 0.88) and moderate specificity (around 0.76). Diagnostic protocols outperformed screening protocols, and machine-learning analysis of short videos showed the strongest reported performance among the modalities assessed in that review. Critically, the authors noted that most evidence came from the United States and called for validation in geographically and demographically diverse populations, including LMICs (28). Studies of caregiver-administered remote assessment further demonstrate feasibility: parents implementing a standardized telehealth infant autism screener achieved high adherence to assessment-fidelity protocols (29), and a scoping review of parent-recorded home videos for remote assessment of children with disabilities found this approach to be a reproducible route to capturing behavior in naturalistic settings (30). Together, these studies suggest that telehealth, especially when combined with caregiver participation and machine-learning analysis of home video, is among the most credible near-term levers for extending early detection to underserved populations.

#### 4.2.5 Diagnostic accuracy, validation gaps, and dataset bias

The enthusiasm warranted by these advances must be tempered by consistent methodological limitations that recur across modalities. Reported accuracies are often obtained on small, curated datasets under controlled conditions and degrade on external validation (12, 13). Datasets are frequently biased toward particular sexes, ethnicities, and geographic regions, restricting generalizability and risking systematically poorer performance for underrepresented groups, an especially serious concern given the well-documented under-recognition of autism in girls and in racial, ethnic, and socioeconomically disadvantaged groups (8, 12, 28). Many studies lack prospective, real-world evaluation, and few compare automated tools head-to-head against the conventional gold standard in the populations where they would actually be deployed. From a public-health perspective, these are not peripheral caveats: a screening tool that performs unevenly across populations can entrench rather than reduce disparities, and external validity, not peak accuracy, should be the primary criterion for adoption.

#### 4.2.6 Equity appraisal

Across secondary prevention, the equity calculus is genuinely promising but conditional. Telehealth and low-cost, app- or video-based AI screening score highly on reach, affordability, and scalability, offering a credible path to identifying children currently missed by specialist-dependent systems (15, 28–30). Wearable approaches can provide continuous and ecologically relevant physiological or behavioral data, whereas biomarker-based approaches may add biological objectivity in selected settings; both nevertheless face important affordability, regulatory, usability, and validation challenges (14, 25–27). The decisive issue for all of them is equity of performance: only deliberate inclusion of diverse populations in development and validation will ensure that these tools narrow rather than widen the autism diagnostic gap.

### 4.3 Intervention, management, and long-term support

Tertiary prevention encompasses interventions and supports intended to optimize functioning, participation, and quality of life across the lifespan. Here, emerging information technologies are used both to deliver therapeutic content directly and to extend the reach of human-delivered intervention, and the evidence base now includes a growing number of randomized trials and meta-analyses.

#### 4.3.1 Digital interventions, mHealth, and digital therapeutics

Digital interventions include computer-, tablet-, smartphone-, and web-based programs, as well as app-supported digital therapeutics and caregiver-facing mobile health tools; mobile-app reviews further highlight usability and software-design considerations as central to real-world adoption (16). These technologies are considered together here because, from a public-health perspective, their shared promise lies in delivering intervention content or coaching support without requiring every contact to occur face to face with a specialist. Several syntheses have attempted to quantify their effectiveness for autism. A 2024 systematic review and meta-analysis of digital interventions for ASD pooled 33 randomized studies involving 1,285 participants and reported significantly greater improvement in overall ASD-related outcomes for digital-intervention groups than for controls. Effects were notably larger when digital interventions were compared with waitlist, treatment-as-usual, or no-treatment comparators, but smaller and non-significant when compared with active controls (31). This pattern is the single most important interpretive caveat in the tertiary-prevention literature: digital tools show clearer benefits when compared with inactive or minimal-intervention controls than when compared with active conventional interventions. The authors also noted the potential of digital delivery to reduce the economic burden of intervention, a key public-health consideration (31).

#### 4.3.2 Immersive technologies: virtual and augmented reality

VR and AR create controlled, repeatable, and engaging environments in which autistic individuals can practice social, communication, cognitive, and motor skills. A 2025 systematic review of VR interventions for social skills in autistic children and adolescents analyzed 14 studies and found generally positive effects, with larger benefits for individuals with stronger baseline cognitive or language abilities and for complex skills delivered through immersive VR, while non-immersive VR offered advantages in cost and flexibility for more basic skills; the review also flagged adverse effects such as dizziness, eye fatigue, and sensory overload, particularly in immersive settings (17). A meta-analysis of randomized controlled trials of immersive VR-based training reported significant pooled benefits for social, emotional, and cognitive skills, while explicitly noting that accessibility, affordability, customization, and cost are decisive considerations for real-world implementation (32). Beyond social cognition, VR has been applied to motor and postural domains; a 2025 randomized controlled trial found that non-immersive VR combined with conventional physiotherapy improved postural balance in autistic children more than physiotherapy alone (33). The collective signal is that immersive technologies are promising adjuncts whose benefits, side effects, and cost-effectiveness depend heavily on the match between the technology (immersive versus non-immersive), the skill targeted, and the individual. In addition, recent reviews of extended-reality design emphasize that autistic users are still inconsistently involved in the design and evaluation of immersive technologies, reinforcing the need for participatory and sensory-informed development (18).

#### 4.3.3 Social and assistive robotics

Socially assistive robots have been studied as engaging, predictable interaction partners that can support social and communication skill development. Robot-assisted autism therapy has been combined with AI methods, such as computer-vision-based detection of engagement and gaze analysis, to make sessions adaptive and to quantify therapeutic dose; pilot work has used metrics such as eye-contact frequency and inter-eye-contact delay to identify optimal session parameters, including for children with co-occurring autism and intellectual disability who are often under-represented in research (34, 35). Controlled comparisons, however, counsel humility about mechanism. A randomized controlled trial comparing dog-assisted therapy with robot-dog-assisted therapy and a no-treatment control found that, at the group level, the live-animal condition produced greater gains in emotional attunement and regulation than either the robot or no-treatment conditions, although individual-level analyses showed that some children responded strongly to the robot (36). Robotics thus illustrates a general theme: technological novelty and engagement do not automatically translate into superiority over established human- or animal-mediated approaches, and individualization may matter more than the modality itself. From an implementation perspective, robotics also raises a sharper scalability question than many other digital interventions. Even when individual children engage well with robots, the cost, maintenance requirements, need for trained facilitators, and uncertain incremental benefit over human-mediated, caregiver-mediated, or animal-assisted approaches limit their near-term relevance as population-scale public-health tools.

#### 4.3.4 Telehealth-delivered intervention and caregiver coaching

Telehealth supports not only assessment but also intervention, frequently by training and coaching caregivers to deliver evidence-based strategies in the home, an approach with strong public-health logic because it multiplies the reach of scarce specialists and embeds intervention in everyday routines. Studies have demonstrated that parents can be trained via telehealth to implement structured behavioral techniques such as discrete-trial training with adequate fidelity (37), and parent-mediated, remotely coached models are increasingly central to scalable service delivery. The World Health Organization’s Caregiver Skills Training program exemplifies how caregiver-mediated, potentially technology-supported models are being promoted globally to extend support where specialist services are absent (9). Caregiver-mediated telehealth is therefore not merely a pandemic-era contingency but a structurally important route to equitable tertiary prevention.

#### 4.3.5 Wearables and continuous management

Within tertiary prevention, wearables shift from a screening role to one of ongoing management: real-time monitoring of physiological arousal can support the detection of stress or impending distress and enable timely, individualized responses by caregivers and clinicians (14, 25). Reviews of wearable sensors for safety during physical activity similarly position these devices as tools for ongoing risk reduction and participation (26). The same limitations identified earlier apply with equal force to management applications. These include comfort and sensory tolerability, individual variability in physiological signatures, data privacy, regulatory status, and the need for real-world validation (14, 25, 26). Continuous monitoring should therefore not be equated with improved care: without validated thresholds, clear response protocols, and safeguards against surveillance burden, wearable systems may generate alerts and data streams without producing meaningful gains in autonomy, participation, or caregiver support.

#### 4.3.6 Generative AI and conversational agents

The newest entrants to tertiary prevention are large language models and other generative-AI systems. Beyond information provision, generative AI is being evaluated for clinical-interviewing and assessment support. A 2025 study of an AI-assisted clinical interview aligned with diagnostic criteria reported that, across several mental-health conditions, including ASD, the system achieved agreement, sensitivity, and specificity comparable to or exceeding established rating scales. Most participants also rated the interview as empathic and supportive, and the authors emphasized scalability, low cost, and positive user experience as reasons such tools might complement traditional methods (38). These results are early and require independent replication. The equity hazards documented for generative AI remain salient, including uneven accuracy across languages and the potential to propagate misinformation (24). Conversational agents may eventually support skill practice, caregiver guidance, and navigation of services, but their role in autism intervention is at present speculative and must be evaluated with the same rigor and equity scrutiny applied to other modalities.

#### 4.3.7 Effectiveness and equity appraisal

Taken together, the tertiary-prevention literature supports cautious optimism. Digital, immersive, robotic, and telehealth-delivered interventions can produce measurable benefits, particularly for functional and adjacent outcomes and relative to no intervention (17, 31–33), and caregiver-mediated telehealth offers a structurally scalable model (9, 37). At the same time, effects on core features are inconsistent, advantages over active conventional therapies are modest or unproven, trials are frequently small and heterogeneous with variable methodological quality and short follow-up, and questions of durability and generalizability are largely unresolved (17, 31, 32, 36). From an equity perspective, tertiary-prevention technologies are most valuable when they extend access to support for families who would otherwise receive little or no specialist care. However, their public-health value depends on affordability, usability, caregiver burden, and integration into routine services. Tools that are costly, difficult to sustain, or poorly adapted to sensory and cultural needs may reproduce the same inequities they are intended to address. The most defensible public-health conclusion is that these technologies are best positioned as complements and extenders of conventional care, especially valuable where conventional care is unavailable, rather than as replacements.

## 5 Cross-cutting public-health analysis

The stage-based synthesis in Section 4 clarifies where and how emerging information technologies are being applied across the autism service continuum. However, several determinants of population-level value operate across technologies and service stages and cannot be adequately assessed within a single stage. This section therefore provides a cross-cutting analysis of the factors that influence whether technological innovation translates into equitable and sustainable public-health benefit.

The analysis focuses on health equity and access, scalability and health-system integration, data privacy and governance, ethics and neurodiversity, regulation and clinical validation, and practical implementation barriers. These themes are considered across technology categories to identify shared opportunities, recurring limitations, and trade-offs between technical performance, accessibility, affordability, specialist requirements, and real-world feasibility. Figure 2 summarizes this cross-cutting pathway from technical performance to equitable public-health benefit.

**Figure 2:**
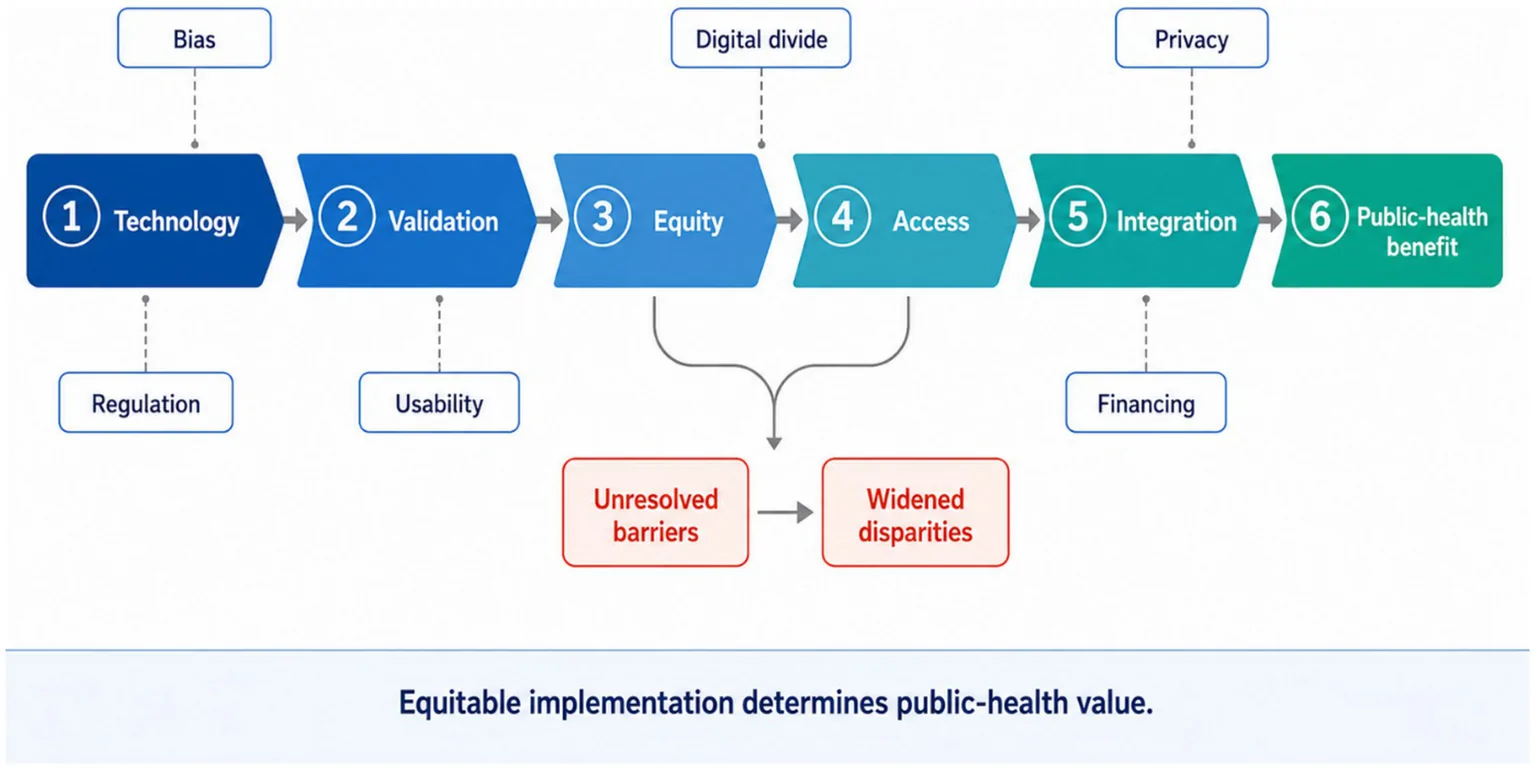
Equity-centered pathway from technology to public-health benefit. Technical performance alone is insufficient for population-level value. Emerging information technologies must be externally validated, equitable in performance, accessible and affordable, and integrated into health systems to produce meaningful public-health benefit. If barriers such as bias, digital exclusion, weak governance, regulatory immaturity, poor usability, and financing gaps are not addressed, these tools may widen rather than reduce disparities.

### 5.1 Health equity and access

The strongest public-health argument for these technologies is their potential to reach populations that specialist-dependent systems miss. Telehealth, low-cost app- or video-based screening, and caregiver-mediated remote intervention could extend identification and support into rural areas and LMICs (15, 28, 29, 37). However, the same tools may widen disparities through unequal access to devices, connectivity, electricity, digital literacy, and language-appropriate content (39). Evidence that AI systems can perform unevenly across linguistic and demographic groups reinforces that equity must be a design requirement rather than a downstream evaluation (20, 21, 24, 40).

### 5.2 Scalability and integration into health systems

Population benefit depends on whether tools can be embedded in primary care, community services, and routine clinical workflows, a challenge widely recognized in implementation frameworks for health and care technologies (41). Implementation-oriented frameworks and review protocols emphasize the importance of understanding for whom, where, and under what conditions hybrid service models may work, rather than focusing solely on average effectiveness (42). Scalable deployment must reduce rather than reproduce dependence on scarce specialists, which is why caregiver-mediated and task-shifted models are especially important (9, 37).

Taken together, the emerging technology ecosystem is better understood as tiered rather than uniform. Community-oriented technologies, including mHealth, app- and video-based screening, telehealth, and remote caregiver support, can extend awareness, initial screening, referral, and intervention beyond specialist centers. In contrast, EEG, biomarker panels, and multimodal assessment may support more complex or confirmatory evaluations but require greater expertise, infrastructure, and financial resources. A tiered service model can connect these complementary technologies according to clinical need and local capacity (Figure 3).

**Figure 3:**
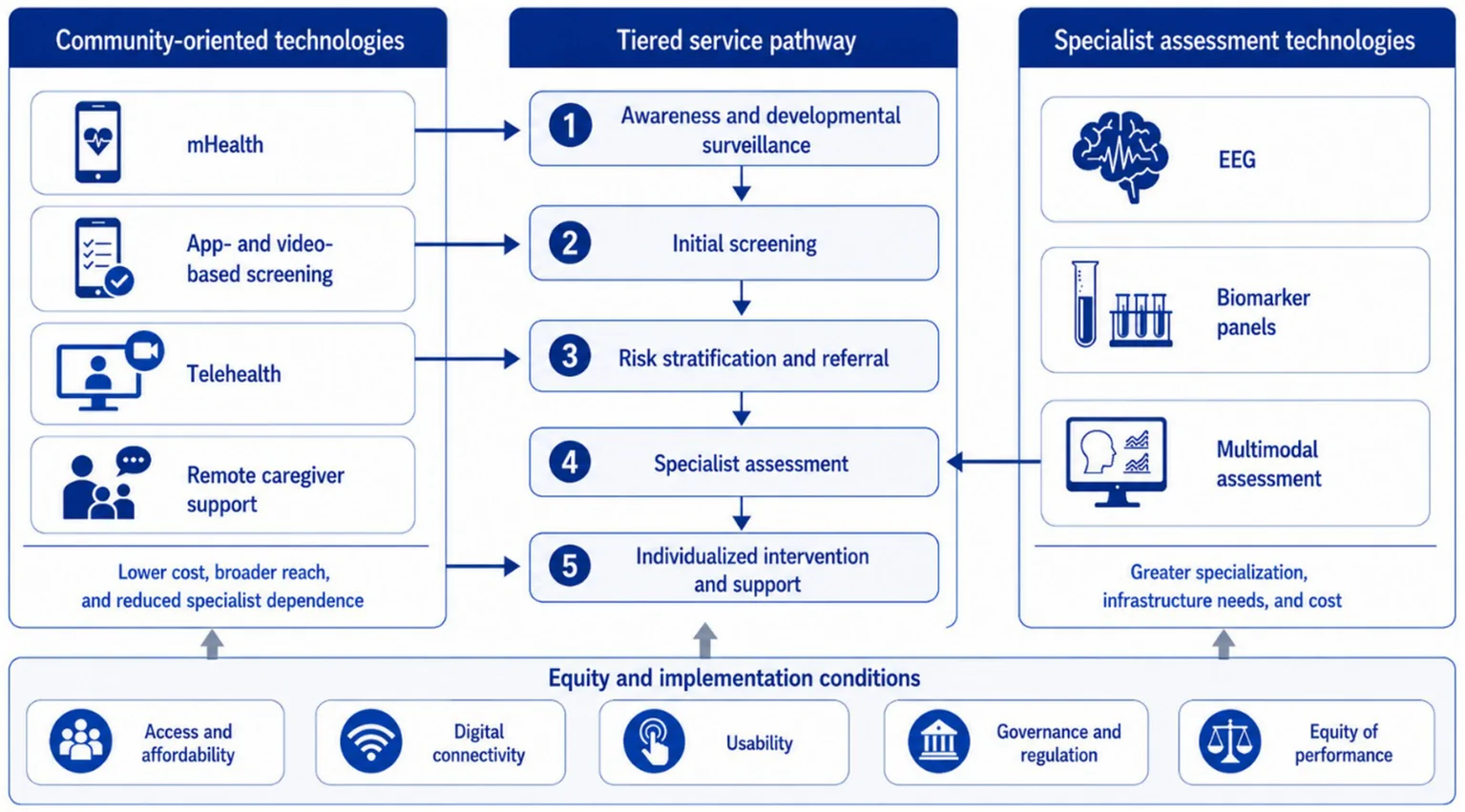
A tiered technology ecosystem for equitable autism service delivery. Community-oriented technologies support awareness, screening, referral, and caregiver-delivered intervention, whereas specialist-oriented technologies may support more complex or confirmatory assessment in selected settings. Equity and implementation conditions influence the effectiveness of the entire pathway. Arrows indicate predominant rather than exclusive roles.

### 5.3 Data privacy, security, and governance

Autism-related digital technologies often collect sensitive data, including child video, physiological signals, behavioral records, developmental histories, and sometimes biomarker or genomic information. Reviews of wearables and healthcare AI repeatedly identify privacy and security as unresolved concerns (25, 26, 43). For minors and people with variable consent capacity, trustworthy deployment requires clear governance for consent, data minimization, storage, secondary use, and family or individual control over data.

### 5.4 Ethics, neurodiversity, and participation

Ethical evaluation extends beyond privacy. Bias in healthcare AI can lead to unequal access, misdiagnosis, and discrimination, making representative data, algorithm audits, transparency, accountability, and explainability essential (20). In autism, technologies should support autistic people’s own goals and wellbeing rather than enforce normative behavior. Autistic individuals and families should therefore be involved in design, evaluation, and governance, especially where tools address early identification, monitoring, or intervention (1, 5, 7, 44, 45).

### 5.5 Regulation and clinical validation

Many tools remain ahead of regulatory and clinical validation. For example, reviews of commercial wearables note that many devices used in autism-related contexts have unclear regulatory status (25). Before public-health adoption, technologies require prospective validation in representative populations, appropriate comparators, and transparent regulatory pathways. Without this, unvalidated tools may generate harm, increase unnecessary referrals, or erode trust.

### 5.6 Implementation barriers

Practical barriers include workforce training, digital literacy, infrastructure, financing, sustainability beyond pilot programs, and reimbursement mechanisms. These factors often determine whether a technically promising tool can become a routine service, rather than being abandoned after small-scale demonstration (41). Public-health planning should therefore treat implementation and sustainability as first-order design considerations rather than as issues to be addressed only after efficacy has been demonstrated (42). Figure 2 synthesizes the central public-health argument of this review: technical performance alone is insufficient, and equitable implementation determines whether emerging information technologies produce population-level benefit.

## 6 Discussion

### 6.1 What the evidence supports across the continuum

Building on the stage-based evidence synthesis in Section 4 and the cross-cutting public-health analysis in Section 5, this section interprets the relative maturity, public-health relevance, and principal trade-offs of the technologies reviewed through a consistent health-equity lens. The original contribution of this review lies less in describing individual technologies, many of which have been reviewed elsewhere, than in re-embedding a fragmented, technology-specific evidence base within a single public-health service continuum. Read through that lens, the field looks different from how it appears in technology-specific reviews, because the decisive question becomes not “how accurate is this tool?” but “for whom, in what settings, and at what cost does it expand access?” With that framing in mind, several conclusions are reasonably robust. First, emerging information technologies are most mature and most directly equity-relevant at the secondary-prevention stage: telehealth and low-cost, app- or video-based AI screening have accumulated the most credible evidence for extending timely identification beyond specialist-dependent systems, with meta-analytic support for video-assisted telehealth diagnosis and demonstrated feasibility of caregiver-administered remote screening (15, 28–30). Second, at the tertiary stage, digital, immersive, robotic, and telehealth-delivered interventions can produce measurable benefits, but chiefly relative to no intervention and for functional or adjacent outcomes; their advantage over active conventional therapy is modest or unproven, and effects on core features are inconsistent (17, 31, 32, 36). Third, primary prevention is the least mature stage, comprising promising but unvalidated risk-prediction models, pre-translational biological markers, and scalable but accuracy-sensitive awareness tools (19, 23, 24). Across all stages, the technologies are best understood as complements and extenders of conventional care, most valuable precisely where conventional care is scarce, rather than as substitutes.

### 6.2 A matrix view: technology by prevention level

A matrix view helps clarify the distribution of technologies across the continuum. AI, telehealth, wearables, and generative AI span multiple stages, whereas immersive technologies and robotics are concentrated mainly in tertiary prevention. Table 1 summarizes each technology category in terms of stage, applications, indicative evidence strength, equity risks, and implementation barriers. The evidence-strength column reflects a qualitative reading of the maturity and consistency of the cited literature rather than a formal grading such as GRADE, and is intended to orient readers rather than to substitute for study-level appraisal.

**Table 1:** Emerging information technologies across the autism service and prevention continuum: applications, evidence, equity risks, and implementation barriers.

Technology	Main continuum stage(s)	Main applications	Indicative evidence strength	Principal equity risks	Dominant implementation barriers
AI/machine learning (including computer vision, speech/NLP, and explainable AI)	Primary to tertiary	Risk prediction from background or medical data (19); behavioral and EEG-based screening/diagnosis (12, 13, 22); engagement analytics in therapy (34, 35)	Moderate for screening, but with wide accuracy ranges and mostly retrospective or curated data (12, 13); low-moderate for risk prediction (19)	Dataset bias by sex, ethnicity, language, and geography; poorer performance for under-represented groups (12, 28)	External validation; dataset representativeness; clinician interpretability and trust
mHealth/digital therapeutics	Primary to tertiary	App- and tablet-based education, screening support, caregiver guidance, and intervention delivery (31); language-sensitive health-information support and associated accuracy risks (24)	Low-moderate; evidence often bundled within broader digital-intervention studies (31)	Unequal smartphone access, connectivity, language availability, and digital literacy	App quality assurance; engagement and adherence; privacy; workflow integration
Wearables/sensors/IoT	Secondary to tertiary	Physiological and behavioral characterization; stress and arousal monitoring; sleep and safety applications (14, 25–27)	Low-moderate; small samples, high inter-individual variability, and sensor-accuracy limits (14, 27)	Device cost, limited access in low-resource settings, and sensory tolerability	Comfort and usability; data privacy; undefined regulatory status (25); real-world validation
Telehealth/telemedicine	Secondary to tertiary	Remote screening and diagnosis (15, 28); caregiver-administered assessment (29, 30); remote parent coaching (37)	Moderate-strong for diagnosis, with meta-analytic support (28); promising for caregiver-mediated delivery (29, 37)	Digital divide in connectivity, devices, and literacy; evidence concentrated in high-income settings (28)	Infrastructure; workforce training; reimbursement and service integration
Immersive technologies (VR/AR)	Tertiary	Social, emotional, cognitive, and motor skill training (17, 32, 33)	Moderate; positive but heterogeneous trials, short follow-up, and uncertain benefit versus active care (17, 32)	Hardware cost and access; benefits may be greater among participants with stronger baseline cognitive or language abilities (17, 32)	Hardware cost; side-effects such as dizziness or sensory overload (17); customization
Social/assistive robotics	Tertiary	Predictable interaction partners for social-communication practice; engagement measurement (34–36)	Low-moderate; small studies and no clear superiority over human- or animal-mediated care (36)	High cost; concentration in well-resourced research settings	Maintenance; trained facilitation; individual response variability (36); service integration
Generative AI/large language models	Primary to tertiary	Health information and education; assessment support; conversational guidance (24, 38)	Very early, promising single studies requiring replication (38)	Uneven accuracy across languages; misinformation risk for linguistic minorities (24)	Validation, safety, and governance; accuracy assurance across populations

### 6.3 Key gaps in the current evidence

Four gaps recur. The first is external validity: much of the evidence rests on small, curated datasets and controlled conditions, with limited prospective evaluation in the populations and settings where deployment is intended (12, 13, 31). The second is representativeness: datasets and trials are skewed toward high-income settings and toward particular sexes and ethnicities, threatening equitable performance and concentrating evidence in countries such as the United States, even for tools whose greatest potential value is in LMICs (9, 28). The third is durability and comparative effectiveness: few studies offer long follow-up or head-to-head comparison against active conventional care (31, 32, 36). The fourth is implementation evidence: data on real-world integration, sustainability, context-specific effectiveness, and long-term service delivery remain limited relative to efficacy-focused research (31, 42).

### 6.4 Tensions for public-health practice

These gaps crystallize into several tensions that public-health decision-makers must navigate. First, there is a tension between specialization and accessibility. More resource-intensive modalities, including neurophysiological and biomarker-based approaches, may offer greater biological or measurement objectivity in selected settings but generally require specialized equipment, technical expertise, and infrastructure, which limits their scalability. In contrast, community-oriented approaches such as smartphone-based screening, video-assisted assessment, and caregiver-administered telehealth can extend reach and reduce dependence on geographically concentrated specialist services, although their performance still requires rigorous external validation across diverse populations and real-world settings (13, 28, 29). Second, there is a tension between innovation and equity: the rapid deployment of consumer technologies and generative AI may outpace clinical validation and potentially widen disparities through unequal digital access and uneven performance across demographic and linguistic groups (20, 24, 25). Third, there is a tension between monitoring and autonomy: risk-oriented and continuous-monitoring technologies should expand access to timely support without contributing to stigmatization, deterministic interpretations, excessive surveillance, or erosion of the autonomy of autistic individuals (5, 7). Navigating these tensions, rather than maximizing any single measure of technical performance, is therefore central to an equity-centered public-health approach.

### 6.5 Limitations of this review

This review has several limitations. First, PubMed was used as the sole searchable bibliographic database. This choice reflected the clinical and public-health orientation of the review and enabled focused coverage of biomedical, developmental, rehabilitation, digital-health, and health-services research. Nevertheless, relevant studies published primarily in engineering, computer science, human–computer interaction, education, and assistive-technology sources may have been underrepresented. The review should therefore be interpreted as a clinically and publicly oriented narrative synthesis rather than an exhaustive multidisciplinary account of all technological research related to autism. Second, the restriction to English-language publications may have resulted in the omission of evidence and implementation experience from non-English-speaking regions, including some low- and middle-income countries. Third, because this was a narrative review, we did not use a preregistered protocol, conduct a formal study-level risk-of-bias assessment, or apply a quantitative certainty-of-evidence framework. Although explicit eligibility criteria and an equity-oriented analytical framework were used to improve transparency and consistency, some degree of selection and interpretive judgment remains unavoidable. Fourth, much of the available evidence focuses on children and caregiver-mediated services, whereas research involving adolescents, adults, and older autistic people remains comparatively limited. Finally, the evidence-strength categories presented in Table 1 represent a qualitative assessment of the maturity, consistency, and limitations of the cited literature rather than a formal grading system such as GRADE. These categories are intended to help readers identify broad patterns of evidence and uncertainty and should not be interpreted as substitutes for study-level methodological appraisal.

## 7 Future directions and recommendations

For research, the priority is rigorous, real-world evidence. Screening and diagnostic tools should be prospectively validated in diverse, representative, and explicitly multinational cohorts. Intervention studies should be larger and longer, include active comparators, and use meaningful functional and quality-of-life outcomes. Transparent reporting should include performance disaggregated by sex, ethnicity, language, and setting, so that equity of performance can be assessed rather than assumed (12, 20, 28, 32). Multimodal approaches that combine behavioral, physiological, and, where validated, biological signals appear promising and should be prioritized over single-modality systems when they improve robustness and equity (12, 23).

For policy, emerging information technologies should be embedded within strengthened systems rather than treated as stand-alone fixes. This means investing in connectivity, devices, and digital literacy; establishing clear regulatory and clinical-validation pathways (25); building data-governance frameworks for populations that include minors and people with variable consent capacity (25, 43); and creating appropriate financing and reimbursement mechanisms to support the sustainable use of validated technologies (42). Caregiver-mediated, task-shifted, and technology-supported models in under-resourced settings deserve particular support (9).

For practice, the guiding principle should be equity-centered, participatory design. Tools should be co-designed with autistic people and their families, using participatory methods adapted to autistic communication preferences and sensory needs, evaluated for usability and sensory tolerability as well as technical performance (14, 25, 44), integrated into clinical and community workflows (11, 42, 43), and positioned to complement and extend human-delivered care rather than replace it. Throughout, the framing should remain one of expanding access and support while avoiding stigmatizing or deterministic uses.

## 8 Conclusion

Autism is a major and growing global public-health concern marked by a profound gap between the interventions known to help and the services most affected people can access (1, 9). Emerging information technologies offer realistic opportunities to narrow this gap, although their contributions differ substantially across technological modalities. Community-oriented approaches such as telehealth and app- or video-based screening may extend reach and reduce dependence on geographically concentrated specialist services, whereas more resource-intensive approaches, including neurophysiological and biomarker-based technologies, may provide complementary forms of assessment or measurement but require greater expertise and infrastructure. These technologies include AI and machine learning, wearables and IoT, mHealth, telehealth, immersive media, robotics, and generative AI. The strongest near-term evidence is at the screening and diagnostic stage, with a growing but more qualified evidence base for intervention (17, 28, 31). Whether these opportunities translate into equitable population-level benefit, however, will depend on external validity, representativeness, affordability, accessibility, ethics, data governance, regulation, health-system integration, and implementation in real-world and resource-limited settings. Treated as complements to, rather than replacements for, conventional care, and designed from the outset around health equity and the priorities identified by autistic people, families, and the wider autism community, emerging information technologies can become valuable components of a more accessible and equitable autism service system across the service and prevention continuum.
